# COVID‐19 disease in professional football players: symptoms and impact on pulmonary function and metabolic power during matches

**DOI:** 10.14814/phy2.15337

**Published:** 2022-06-14

**Authors:** Chiara Gattoni, Emanuele Conti, Andrea Casolo, Stefano Nuccio, Carmine Baglieri, Carlo Capelli, Michele Girardi

**Affiliations:** ^1^ Institute of Orthopaedics and Musculoskeletal Science University College London Royal National Orthopaedic Hospital Stanmore UK; ^2^ 2591 School of Sport, Rehabilitation and Exercise Sciences University of Essex Colchester UK; ^3^ 9308 Department of Biomedical Sciences University of Padua Padua Italy; ^4^ Department of Movement, Human and Health Sciences University of Rome “Foro Italico” Rome Italy; ^5^ 19051 Department of Neuroscience, Biomedicine and Movement Sciences University of Verona Verona Italy

**Keywords:** fatigue, soccer, long COVID‐19, muscle fatigue, performance, spirometry

## Abstract

This study aimed at: (1) Reporting COVID‐19 symptoms and duration in professional football players; (2) comparing players’ pulmonary function before and after COVID‐19; (3) comparing players’ metabolic power (P_met_) before and after COVID‐19. Thirteen male players (Age: 23.9 ± 4.0 years, V̇O_2peak_: 49.7 ± 4.0 mL/kg/min) underwent a medical screening and performed a running incremental step test and a spirometry test after COVID‐19. Spirometric data were compared with the ones collected at the beginning of the same season. Players’ mean P_met_ of the 10 matches played before COVID‐19 was compared with mean P_met_ of the 10 matches played after COVID‐19. Players completed a questionnaire on COVID‐19 symptoms and duration 6 months following the disease. COVID‐19 positivity lasted on average 15 ± 5 days. “General fatigue” and “muscle fatigue” symptoms were reported by all players during COVID‐19 and persisted for 77% (general fatigue) and 54% (muscle fatigue) of the players for 37 ± 28 and 38 ± 29 days after the disease, respectively. No significant changes in spirometric measurements were found after COVID‐19, even though some impairments at the individual level were observed. Conversely, a linear mixed‐effects model analysis showed a significant reduction of P_met_ (−4.1 ± 3.5%) following COVID‐19 (*t* = −2.686, *p* < 0.05). **“**General fatigue” and “muscle fatigue” symptoms may persist for several weeks following COVID‐19 in professional football players and should be considered for a safer return to sport. Players’ capacity to compete at high intensities might be compromised after COVID‐19.

## Graphical abstract

“General fatigue” and “muscle fatigue” symptoms were the most common long‐term COVID‐19 symptoms reported. Players’ capability to exercise at high intensity was compromised following COVID‐19. Players’ pulmonary function was not affected by COVID‐19, even though some impairments were observed at the individual level.

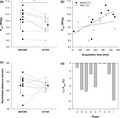

## 1 INTRODUCTION

The severe acute respiratory syndrome coronavirus‐2 (SARS‐CoV‐2) began to spread globally in early 2020, causing an infectious disease of pandemic proportions, called COVID‐19. COVID‐19 symptoms can be highly heterogeneous among individuals. In the athletes population a prevalence of mild or no symptoms have been observed, lasting approximately 1 week/10 days with no need for hospitalization (Schumacher et al., 2021; Schwellnus et al., 2021). However, differently from clinical populations where the type and duration of COVID‐19 symptoms have extensively been reported (Huang et al., 2021; Huang, Wang, et al., 2020; Huang, Lian, et al., 2020), more limited data are available on athletes (Hull et al., 2022; Petek et al., 2021; Schwellnus et al., 2021). As a result, very little is known about the clinical pattern, symptom time course, and long‐term impact of COVID‐19 in this population, raising uncertainty about how athletes can safely return to sport (RTS).

One of the main goals of any RTS program is to help athletes regain their capacity to train and compete at high intensities, and this is particularly relevant at professional levels. In general, the process of RTS decision‐making is essential to balance the negative effects of detraining on athletes’ fitness level, against the potential risks associated with the post‐disease/injury sequelae (Hull et al., 2022; Stokes et al., 2020). Unfortunately, in the context of COVID‐19, these risks are still largely unknown. Indeed, despite some guidelines for a safe RTS following COVID‐19 have recently been published (Hughes et al., 2020; Kim et al., 2021; Phelan et al., 2020; Rico‐González et al., 2021; Sprouse et al., 2020; Wilson et al., 2020), there is very limited knowledge on the post‐disease sequelae in the athletes population, challenging sport scientists and physicians RTS decision‐making.

“General fatigue” and “muscle fatigue” are among the most common perceived symptoms documented during and after COVID‐19 (Huang et al., 2021). However, the number of studies investigating type and duration of these symptoms in the athletes population is still insufficient (Hull et al., 2022; Petek et al., 2021; Schumacher et al., 2021; Schwellnus et al., 2021). Fatigue symptoms could be of particular relevance in the process of RTS decision‐making, as they may negatively affect athletes’ performance, as well as increase the risk of injuries and other negative outcomes following COVID‐19. To the best of our knowledge, only one study has ever compared physical performance before and after COVID‐19 in athletes, showing decrements in peak power output during an incremental cardiopulmonary exercise test (Fikenzer et al., 2021). Nevertheless, future studies are needed to confirm these results and shed some light on the long‐term COVID‐19 symptoms affecting athletes.

Other COVID‐19 symptoms that should be considered for a safer RTS are those related to the lower respiratory tract (e.g., chest pain, dyspnoea and cough), as they seem to be associated with prolonged disease and delayed RTS (Hull et al., 2022) and to affect pulmonary function (Fikenzer et al., 2021). There is limited and contradictory data on the effect of COVID‐19 on pulmonary function in the athletes population. Specifically, some studies have reported impairments in some of the spirometry measures tested following COVID‐19 (Fikenzer et al., 2021; Komici et al., 2021; Milovancev et al., 2021), while others have not (Gervasi et al., 2021; Komici et al., 2021). Different factors may have accounted for such discrepancies including, different types of research design used and athletes’ fitness level (Fikenzer et al., 2021; Gervasi et al., 2021; Komici et al., 2021; Milovancev et al., 2021). It is also worth noting that it is still unclear whether spirometry tests may be of prognostic value or should be used as predictors of respiratory symptoms following COVID‐19 in athletes.

The aims of the present study were to: (1) report type and duration of COVID‐19 symptoms in 13 professional football players; (2) quantify the impact of COVID‐19 on performance by comparing players’ metabolic power (P_met_, W/kg) during 10 official matches played before COVID‐19 and 10 official matches played after COVID‐19; (3) compare players’ pulmonary function before and after COVID‐19.

## 2 MATERIALS AND METHODS

### 2.1 Participants and general overview

This was a retrospective study involving 13 professional football players (see Table 1 for players’ characteristics). This number corresponds to the total number of players within the same team (Italian third division) who contracted COVID‐19 during the 2020/2021 season.

**TABLE 1:** Anthropometric and physiological characteristics of the football players following COVID‐19

	*n* = 13
Age (years)	23.9 ± 4.0
Height (m)	1.83 ± 0.04
Body mass (kg)	78.1 ± 4.8
BMI (kg/m^2^)	23.3 ± 1.2
V̇o_2peak_ (mL/kg/min)	49.7 ± 4.0
HR_peak_ (beats/min)	186 ± 15
SpO_2peak_ (%)	97.7 ± 1.3

Abbreviations: BMI, body mass index; HR_peak_, peak heart rate; SpO_2peak_, oxygen saturation at V̇O_2peak_; V̇O_2peak_, peak oxygen uptake.

The disease was identified through the use of the rhino pharyngeal swab for SARS‐Cov‐2 RNA and the PCR method with AllPlex‐Seegene CE‐IVD (Seegen, Seoul, Republic of Korea). After the first negative post‐COVID‐19 test result, players underwent a series of physical examinations and a comprehensive health‐related screening with the medical staff. Subsequently, players performed a blood test to assess the presence of IgC class anti‐COVID‐19 antibodies (m2000 SARS‐CoV‐2 assay, Abbott Laboratories, Illinois, USA), a spirometry test and a running incremental step test. Spirometry data were compared with the ones collected at the beginning of the same season.

Global Positioning System (GPS) data recorded during the 10 official matches played immediately after COVID‐19 were compared with the GPS data recorded during the 10 official matches played before the disease. Finally, a questionnaire on COVID‐19 symptoms and comorbidities was provided a posteriori.

This study was approved by the Ethics Committee from the University of Essex (ETH2122‐0111), and all the procedures were conducted in conformity with the Declaration of Helsinki. All players signed a written informed consent and agreed to share their data for research purposes. All the tests and procedures used were in accordance with the COVID‐19 RTS guidelines established by the Medical Commission of the Italian Football Federation.

### 2.2 Incremental step test

A running incremental step test for peak oxygen uptake (V̇O_2peak_) determination was performed immediately after the disease on a motorised treadmill (RL3500; Rodby Innovation AB, Vänge, Sweden). During the test, running speed was increased by 1 km/h every 1 min (until volitional exhaustion) starting from 8 km/h. Prior to testing, the players were required to do a 3‐min warm‐up at 4.5 km/h. Pulmonary gas exchange measurements were collected breath‐by‐breath throughout the entire test (Quark CPET, COSMED, Rome, Italy). V̇O_2peak_ was measured as the highest value of a 30‐s moving average. Heart rate (HR) was also collected during the entire duration of the test. HR peak (HR_peak_) was considered as the highest value reached during the test. Oxygen saturation (SpO_2_) was measured immediately after the end of the test (Xpod model, COSMED, Rome, Italy).

### 2.3 Spirometry test

A spirometry test was performed at the beginning of the season (i.e., July 2020) and immediately after COVID‐19 using a Pony FX spirometer (COSMED, Rome, Italy). Players were asked to complete three acceptable forced maximal flow‐volume maneuvres. Forced Expiratory Volume in 1 s (FEV_1_), Peak Expiratory Flow (PEF), Forced Vital Capacity (FVC) and FEV_1_/FVC ratio (FEV_1_/FVC) were recorded in accordance with the ATS/ERS 2005 Guidelines (Miller et al., 2005). These measures were also expressed as percentages of theoretical values, as described elsewhere (Quanjer et al., 1993).

Only 10 players were considered for the analyses as the remaining three joined the football Club during the second half of the season and performed only one spirometry test (after COVID‐19).

### 2.4 GPS data

A portable 50‐Hz GPS system (K‐Sport Universal srl, Pesaro, Italy) was used to compare players’ mean P_met_ (W/kg) during the ten matches played before and the ten matches played after COVID‐19. The GPS device was activated 15 min before the data collection and tested for a correct acquisition satellite signal. P_met_ and distance covered were computed using the Dynamix software (K‐Sport Universal srl, Pesaro, Italy), according to the formulas proposed by Osgnach and colleagues (Osgnach et al., 2016). The mean P_met_ of 11 ± 6 half‐times (45 min) played before COVID‐19 was compared with the mean P_met_ of 10 ± 4 half‐times played after COVID‐19. The distances recorded were normalised by the GPS acquisition time and also compared.

Only nine players were considered for this comparison. Data from the other players were not available either due to some technical issues occurred during the data acquisition or the role played (i.e., one player was the goalkeeper of the team).

### 2.5 COVID‐19 symptoms and comorbidities questionnaire

Players completed a questionnaire on symptoms and comorbidities approximately six months after COVID‐19 (see Table 2). The questionnaire included a list of the most common COVID‐19 symptoms (Hull et al., 2022; Schwellnus et al., 2021), and cardiorespiratory/metabolic diseases. Players indicated symptoms and comorbidities experienced both during and after COVID‐19, specifying their duration. Players had the possibility to report other symptoms and diseases if not already included in the list.

**TABLE 2:** Football players comorbidities and COVID‐19 symptoms and duration

		Cases			
		*n* = 13			
		During COVID−19 *n* (%)	Symptoms duration X̅ ± SD *M*, [min, max]	After COVID−19 *n* (%)	Symptoms duration X̅ ± SD *M*, [min, max]
Comorbidities	No comorbidities	12 (92%)	/	12 (92%)	/
	Asthma	1 (8%)	/	1 (8%)	/
Symptoms	General fatigue	13 (100%)	12 ± 6 11, [5, 24]	10 (77%)	37 ± 28 25, [10, 90]
	Muscle fatigue	13 (100%)	12 ± 6 11, [5, 24]	7 (54%)	38 ± 29 30, [10, 90]
	Muscle pain	11 (85%)	10 ± 4 10, [5, 17]	3 (23%)	37 ± 21 30, [20, 60]
	Headache	9 (69%)	5 ± 2 5, [3, 10]	2 (15%)	4 ± 2 4, [2, 5]
	Anosmia/dysgeusia	8 (62%)	10 ± 3 10, [5, 16]	3 (23%)	13 ± 6 10, [10, 20]
	Fever	6 (46%)	3 ± 2 3, [1, 7]	0 (0%)	/
	Sore throat	6 (46%)	5 ± 2 5, [1, 7]	1 (8%)	3
	Sleep problems	4 (31%)	10 ± 7 11, [3, 17]	2 (15%)	90 ± 42 90, [60, 120]
	Dry cough	4 (31%)	9 ± 4 7, [7, 14]	1 (8%)	20
	Chest pain	3 (23%)	6 ± 1 5, [5, 7]	0 (0%)	/
	Dyspnoea	1 (8%)	16	2 (15%)	100 ± 28 100, [80, 120]
	Chesty cough	1 (8%)	7	0 (0%)	/
	Diarrea	1 (8%)	5	0 (0%)	/
	Abdominal pain	0 (0%)	/	0 (0%)	/
	Muscle damage	0 (0%)	/	1 (8%)	30
	Nausea	0 (0%)	/	0 (0%)	/

Abbreviations: %, percentage of the total number of cases; *M*, median; [min, max], minimum and maximum values (range); SD, standard deviation; X̅ , mean value. Symptoms duration in days.

### 2.6 Statistical analysis

Normality of the data was checked using the Shapiro–Wilk test, histograms, Q‐Q plots and boxplots. As all data were normally distributed, parametric paired *t*‐tests were used to analyse differences in spirometry and GPS measurements before and after COVID‐19.

The associations between the difference in spirometry values before and after COVID‐19 (i.e., ΔFVC, ΔFEV_1_, ΔFEV_1_/FVC, and ΔPEF) and the days of positivity were investigated using the Pearson’s correlation coefficient (*r*). The association between the difference in P_met_ values before and after COVID‐19 (ΔP_met_) and the days of positivity, as well as, the association between the P_met_ percentage difference (%ΔP_met_) and the duration of “general fatigue” post‐infection (days) were also investigated using *r*.

Since the total acquisition time collected before and after COVID‐19 was different for some players, a linear mixed‐effects model (LMM) analysis, with the acquisition time used as a covariate, was also conducted. This analysis was done to reduce the risk of Type 1 error (Hecksteden et al., 2021). Specifically, P_met_ was used as the dependent variable, pre‐ and post‐measures (T1vsT2) and acquisition time (AcqTime) as independent variables, and players were treated as the random variable. The model specification was as follow, P_met_ ~T1vsT2 + AcqTime + (1|individuals). Visual inspection of the residual plots did not reveal any obvious deviations from homoscedasticity or normality.

The Rstudio software (v1.4.1106, PBC, Boston, MA) and R statistical package *"lme4"* were used to perform the LMM analysis (Bates et al., 2014). The SPSS statistical package (version 23.0; SPSS, Chicago, IL) was used for all the other analyses. Statistical significance was accepted at *p *< 0.05 level. All data are presented as means ± SD, unless otherwise stated.

## 3 RESULTS

Thirteen out of 30 football players (i.e., the full team) were found positive to the COVID‐19 test. On average, SARS‐CoV‐2 positivity duration was 15 ± 5 days (Median (*M)* = 16; range = [8, 24]). Players returned to train two/four days after the first negative post‐COVID‐19 test. An average IgC value of 19.41 ± 28.43 AU/mL (*M* = 8.75; range = [1.9, 96.5]) was found, with blood samples collected within 8 and 24 days from the onset of COVID‐19.

Football players general characteristics and cardiorespiratory responses collected during the incremental step test are shown in Table 1. No cardiopulmonary abnormalities were observed during the test. Players’ comorbidities and COVID‐19 symptoms and their duration are shown in Table 2. All players reported some mild symptoms during COVID‐19; players also reported some long‐term symptoms (see Table 2). Seven players experienced post‐infection symptoms for a period longer than 28 days, and three players for a period longer than 84 days.

### 3.1 GPS data

**FIGURE 1:**
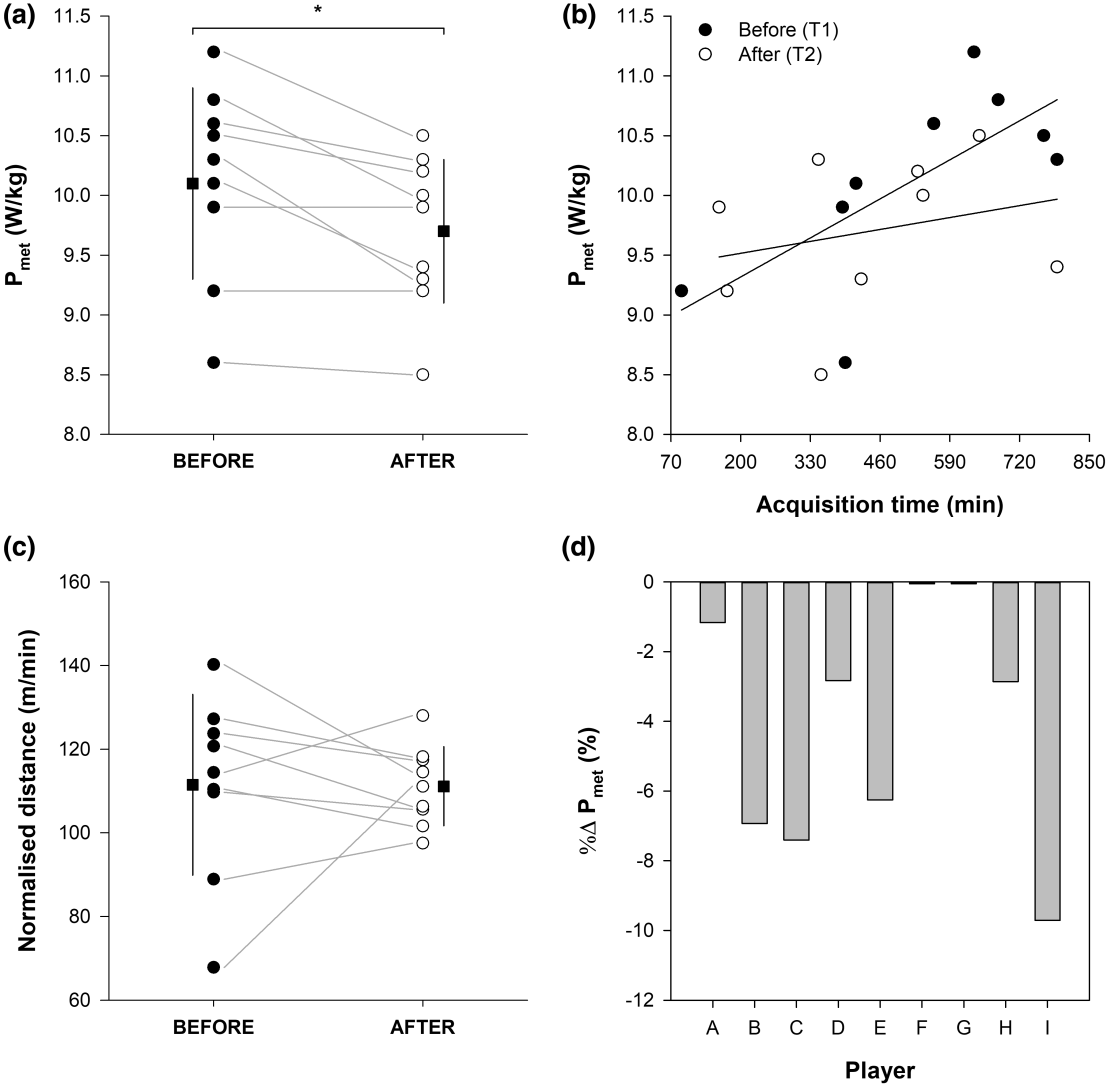
Metabolic power (P_met_) absolute mean values (panel a), P_met_ % changes (panel d), and normalised distance covered (distance covered/acquisition time) before (filled circle) and after (open circle) COVID‐19. Data points depict single players’ values, and the horizontal grey lines help to visualise the changes before and after COVID‐19. Letters in panel d (A‐I) represent the single players. The filled squares indicate the mean values before (on the left‐hand side) and after (on the right‐hand side) COVID‐19, whilst the vertical black lines indicate the standard deviations of the related mean values. P_met_ as a function of the acquisition time before (T1) and after (T2) COVID‐19 is depicted in panel b. Panel b also displays the regression lines for data points before and after COVID‐19. *p < 0.05, before versus after COVID‐19.

A significant decrease of P_met_ in the 10 matches played following COVID‐19 (9.7 ± 0.6 W/kg) compared to the 10 matches played before COVID‐19 (10.1 ± 0.8 W/kg) was observed (*t* = 3.474, *p* < 0.01, Cohen’s *d* = 1.158) (Figure 1). Seven and four out of nine players experienced long‐term “general fatigue” and “muscle fatigue” symptoms after COVID‐19, respectively.

A P_met_ reduction was also confirmed by the LMM analysis, which showed a significant main effect of T1vsT2 (β = −0.387, SE = 0.144, *t* = −2.686, *p* < 0.05), and a non significant main effect of AcqTime (β = 0.0006, SE = 0.0006, *t* = 1.006, *p* = 0.337).

Both the total acquisition time (before COVID‐19: 524 ± 224 min, after COVID‐19: 440 ± 209; *t* = 1.143, *p* = 0.286, Cohen’s *d* = 0.381) and the total distance covered (before COVID‐19: 62045 ± 31579 m, after COVID‐19: 48623 ± 23347 m; *t* = 1.459, *p* = 0.183, Cohen’s *d* = 0.486) did not statistically differ. The distance covered normalised by the acquisition time was also not statistically different (before COVID‐19: 111.4 ± 21.6 m/min, after COVID‐19: 111.1 ± 9.5 m/min; *t* = 0.047, *p* = 0.964, Cohen’s *d* = 0.016) (Figure 1).

No association between ΔP_met_ and days of positivity (*r* = 0.579, *p* = 0.102) and between %ΔP_met_ and duration of “general fatigue” symptom post‐infection (*r* = 0.305, *p* = 0.506) were found.

### 3.2 Spirometry test

**FIGURE 2:**
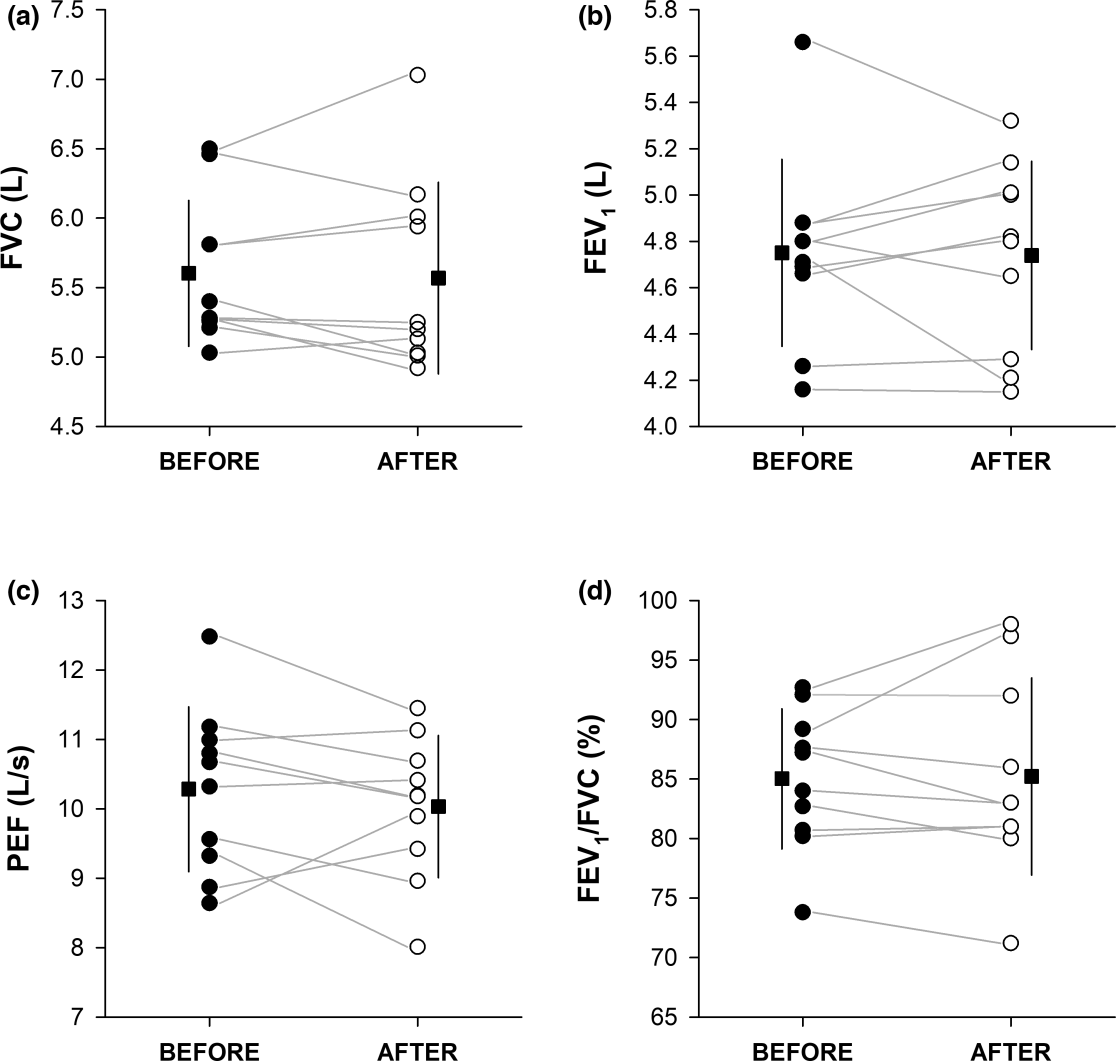
Forced Vital Capacity (FVC, panel a), Forced Expiratory Volume in 1 s (FEV_1_, panel b), Peak Expiratory Flow (PEF, panel c), and FEV_1_/FVC ratio (FEV_1_/FVC, panel d) before (filled circle) and after (open circle) COVID‐19. Data points depict single players’ values, and the horizontal grey lines help to visualise the changes between before and after COVID‐19. The filled squares indicate the mean value before (on the left‐hand side) and after (on the right‐hand side) COVID‐19, whilst the vertical black lines indicate the standard deviations of the related mean values.

Individual absolute values of pulmonary function parameters before and after COVID‐19 are shown in Figure 2. Paired *t*‐tests revealed no significant impairments on pulmonary function parameters (Table 3). No association between ΔFVC (*r* = 0.054, *p* = 0.881), ΔFEV_1_ (*r* = −0.297, *p* = 0.404), ΔFEV_1_/FVC (*r* = −0.339, *p* = 0.337), ΔPEF (*r* = −0.108, *p* = 0.764) and days of positivity was found.

**TABLE 3:** Football players pulmonary function parameters at the beginning of the season (Before COVID‐19) and following COVID‐19 (After COVID‐19)

	Cases			
	*n* = 10			
	Before COVID−19	After COVID−19	∆% COVID−19	*t*‐, *p*‐value, Cohen’s *d*
FVC (L) *M*, [min, max]	5.60 ± 0.53 5.34, [5.03, 6.50]	5.57 ± 0.69 5.22, [4.92, 7.03]	−0.77 ± 4.80 −0.95, [−6.85, 8.15]	*t* = 0.380 *p* = 0.712 *d* = 0.120
FVC_(The)_ (%) *M*, [min, max]	107.22 ± 11.45 106.34, [94.02, 127.95]	106.69 ± 15.37 103.23, [91.79, 138.39]		
FEV_1_ (L) *M*, [min, max]	4.75 ± 0.40 4.76, [4.16, 5.66]	4.74 ± 0.41 4.81, [4.15, 5.32]	−0.13 ± 5.05 1.52, [−10.62, 5.33]	*t* = 0.140 *p* = 0.891 *d* = 0.044
FEV_1(The)_ (%) *M*, [min, max]	110.90 ± 6.38 111.29, [101.46, 123.04]	110.76 ± 8.87 113.81, [92.94, 120.14]		
FEV_1_/FVC (%) *M*, [min, max]	85.02 ± 5.89 85.60, [73.80, 92.70]	85.22 ± 8.29 83.00, [71.20, 98.00]	0.11 ± 4.23 −0.65, [−4.82, 8.74]	*t* = ‐ 0.170 *p* = 0.869 *d* = 0.053
FEV_1(The)_/FVC_(The)_ (%) *M*, [min, max]	104.03 ± 7.09 105.87, [89.91, 112.71]	104.25 ± 9.71 102.61, [86.74, 119.15]		
PEF (L ∙ s^−1^) *M*, [min, max]	10.28 ± 1.19 10.50, [8.64, 12.48]	10.03 ± 1.03 10.19, [8.01, 11.45]	−2.04 ± 8.07 −4.49, [−14.06, 14.47]	*t* = 1.034 *p* = 0.328 *d* = 0.327

Abbreviations: ∆%, percentage difference compared to pre‐COVID‐19 value; FEV_1_, forced expiratory volume in 1 s; FEV_1(The)_, FEV_1_ in percentage respect to the theoretic value; FVC, Forced Vital Capacity; FVC_(The)_, FVC in percentage respect to the theoretic value; L, liters; L ∙ s^−1^ , Liters per seconds; [min, max], minimum and maximum value (range); *M*, median; PEF, peak expiratory flow.

## 4 DISCUSSION

The present study aimed at deepening our knowledge on the effects and symptoms of COVID‐19 in the athletes population. Specifically, we assessed whether the SARS‐CoV‐2 infection would affect the pulmonary function of mildly symptomatic professional football players as well as their physical performance during official matches. Overall, our results showed significant performance (P_met_) decrements during official matches, and no significant changes in pulmonary function (i.e., FEV_1_, PEF, FVC, and FEV_1_/FVC), despite some individual impairments were observed.

### 4.1 COVID‐19 symptoms and duration

In line with previous studies (Al Kuwari et al., 2020; Hull et al., 2022; Schumacher et al., 2021; Schwellnus et al., 2021), all players had mild symptoms and experienced “general fatigue” and “muscle fatigue” during the period of positivity, which lasted on average 15 days. The 77% and 54% of the players also continued experiencing “general fatigue” and “muscle fatigue” symptoms for an average period of 37 and 38 days following COVID‐19, respectively. Other symptoms, such as “muscle pain”, “headache”, “fever”, and “anosmia”/“dysgeusia” were also reported, but in a less percentage (see Table 2). This data confirms mild symptomaticity among young athletes without comorbidities, even though the viral load was not measured and may have significantly influenced and determined the severity of COVID‐19 (Bullard et al., 2020; Schumacher et al., 2021).

Investigating COVID‐19 symptoms in the athletes population is of particular relevance for a safe RTS following the disease. Unfortunately, at the moment there is not enough knowledge on the topic and exhaustive guidelines on RTS decision‐making are not available (Kim et al., 2021; La Scola et al., 2020; Rico‐González et al., 2021). The current study provides further data to the existing literature with the purpose of better describing and characterizing COVID‐19 symptoms in athletes and therefore helping sport scientists and physicians to develop valid protocols to adopt for a safer RTS. Previous studies found that some clusters of COVID‐19 symptoms can potentially predict infections (Menni et al., 2020) and other clinical events in the general population (Lochlainn et al., 2020; Sudre et al., 2021). Similarly, in the athletes population, a specific cluster of COVID‐19 symptoms was found to be directly associated with longer RTS (Schwellnus et al., 2021). The symptoms identified were “excessive fatigue”, “chills”, “fever”, “headache”, “altered/loss sense of smell”, “chest pain/pressure”, “difficulty in breathing”, and “loss of appetite”, with “excessive fatigue” being the symptom associated with the longest RTS and a 70% lower probability of RTS within 40 days from the onset of the symptoms (Schwellnus et al., 2021). In our study, the fatigue symptoms were the only ones affecting all players during the whole COVID‐19 positivity period, and many of them even after (general fatigue: 77%; muscle fatigue: 57%). This would confirm Schwellnus and colleagues’ results (Schwellnus et al., 2021), and strengthen the assumption that fatigue may be a COVID‐19 key symptom to carefully consider when making RTS‐related decisions.

It is worth noting that only a low prevalence of persistent (i.e., >3 weeks from symptoms onset) and exertional cardiopulmonary symptoms were found in a large cohort of US collegiate athletes (1.2% and 4.0%, respectively), with only 0.06% of them experiencing symptoms for periods longer than 12 weeks (Petek et al., 2021). However, concerns were raised for those athletes who experience exertional cardiopulmonary symptoms when returning to sport, especially chest pain, as they might be exposed to an increased risk of cardiac involvement (Petek et al., 2021). These data collectively suggest that, on a large scale, athletes may present a low incidence of developing persistent symptoms following SARS‐CoV‐2 infection. Nevertheless, in the present study, which is in line with a previous report (Hull et al., 2022), a higher occurrence of post‐infection symptoms lasting more than 28 days was observed (see Table 2). Different factors may account for such differences including, sample size, study design, and athletes characteristics. In particular, it is important to highlight that in our study only a small sample of professional football players was tested, which is not necessarily representative of the whole athletes population, where a lower incidence of post‐infection symptoms might be observed. Therefore, further studies involving larger samples of athletes from different sports are essential to elucidate this point.

### 4.2 COVID‐19 and metabolic power

The COVID‐19 pandemic raised serious concerns in the world of sport. In order to prevent the effects of detraining induced by the different lockdowns set worldwide (Girardi et al., 2020), coaches and sports practitioners were forced to find new and alternative training solutions for their athletes (Girardi et al., 2020; Pedersen et al., 2021). However, although some detraining‐induced detrimental effects on physical performance have been documented (Girardi et al., 2020), the effects of COVID‐19 and its combined effects with detraining have not yet been examined.

To the best of our knowledge, our study was among the first ones investigating the effect of COVID‐19 on physical performance (Fikenzer et al., 2021). The results showed that football players expressed significantly lower metabolic power in the 10 matches played immediately after COVID‐19 compared to the 10 matches played immediately before, despite covering the same distance per time. Specifically, players reduced their match intensity by 4.1% ± 3.5% after COVID‐19. These findings confirm and extend the results of Fikenzer and colleagues (Fikenzer et al., 2021), who observed performance impairments in male elite handball players after COVID‐19. Additionally, we observed that “general fatigue” and “muscle fatigue” symptoms were perceived by several players (77% and 54%, respectively) even following COVID‐19, suggesting that these symptoms may have played a key role in affecting players’ performance. Considering that these symptoms seem to be associated with longer RTS (i.e., >40 days) (Schwellnus et al., 2021), and that the players returned to compete on average 19 days after the onset of the infection, the present data suggest that longer RTS would have been more appropriate.

It is important to underline that it is unclear to what extent COVID‐19 and its associated long‐term symptoms influenced players’ performance and to what extent detraining did. Furthermore, another important factor that needs to be considered is that, due to the football federation rules and the spread of COVID‐19 within teams, the matches played following COVID‐19 took place within a shorter period of time (i.e., 35 days), compared to the matches played before (i.e., 68 days). Consequently, a fatigue status might also have occurred for this reason. Further studies are essential to better understand the impact of COVID‐19 on physical performance.

### 4.3 COVID‐19 and pulmonary function

Spirometry data showed overall slight reductions of FVC, FEV_1_, and PEF after COVID‐19, which however did not significantly differ from the values collected at the beginning of the season. Moreover, no association between days of infection and ΔFVC, ΔFEV_1_, ΔFEV_1_/FVC, and ΔPEF was observed, even though the small sample size tested (*n* = 10) and a narrow range of both dependent and independent variables may have hindered some associations. These findings suggest that COVID‐19 does not considerably affect FVC, FEV_1_, FEV_1_/FVC, and PEF in athletes with mild symptoms, which is in line with previous investigations (Fikenzer et al., 2021; Milovancev et al., 2021), but in contrast with others (Gervasi et al., 2021; Komici et al., 2021). No evidence of spirometric impairments was also observed in a large cohort of healthy young adults who had mild‐to‐moderate COVID‐19 symptoms (Mogensen et al., 2022), suggesting that spirometric impairments would unlikely occur in young and healthy populations.

Nevertheless, it is important to highlight that, although no statistical differences were found, some reductions at the individual level were observed after COVID‐19. This is particularly relevant because it reveals that reductions in pulmonary function following COVID‐19 may also occur in some athletes with mild symptoms and potentially delay their RTS. These changes, however, may have also been induced by the period of exercise restriction (i.e., detraining) that athletes had to face during COVID‐19, rather than being directly caused by the virus itself. In line with this, Gervasi and colleagues (Gervasi et al., 2021) found that most of the spirometric parameter reductions observed after COVID‐19 were regained following one month of retraining.

It is worth noting that the lack of abnormalities or impairments found in the spirometric measures does not necessarily imply the absence of any COVID‐19 effects on players’ lung functions, as this test may not be able to detect other levels of impairment. For instance, Lerum and colleagues found that 25% of the individuals examined (*n* = 103) presented structural lung abnormalities and a reduced lung diffusion capacity for carbon monoxide three months after their hospital admission for COVID‐19, without showing substantial impairments in the spirometry test (Lerum et al., 2021). Similar findings were observed in another study (Thomas et al., 2021), even though the post‐infection pulmonary sequelae seemed to be related to the severity of the disease (Thomas et al., 2021). Therefore, caution is recommended when sports scientists and physicians interpret spirometric data, as they may not offer a comprehensive picture of lung health. Nevertheless, future investigations are required to clarify whether COVID‐19 may affect lung structures and functions at other levels in the athletes population, and whether spirometric measures may be helpful from a prognostic perspective.

### 4.4 Study limitations and methodological considerations

A direct comparison with the players of the team who did not contract COVID‐19 would have helped to better identify the impact of COVID‐19 on performance. However, this comparison was not possible due to the lack of data available within this group. Indeed, whereas the players in the COVID‐19 cluster were the main starting players, those in the non‐COVID‐19 cluster were substitutes who often did not even play.

The small sample size tested and the reduced amount of crossed data available when performing the correlation analysis between %ΔP_met_ and the duration of “general fatigue” post‐infection (*r* = 0.305, *p* = 0.506), could have hidden an association. For the same reason, we were unable to investigate the association between P_met_ decrements and other post‐infection symptoms and duration. Further powered studies are required to test any potential relationship between COVID‐19 symptoms and physical performance, in particular those symptoms which seem to be associated with prolonged illness and delayed RTS, such as lower respiratory tract symptoms (Hull et al., 2022) and fatigue (Schwellnus et al., 2021).

Lastly, the severity of long‐term COVID‐19 symptoms was not evaluated. This could have provided more insights into the effects of long‐term COVID‐19 symptoms on performance. Future studies are needed to better understand the impact of acute‐ and long‐term COVID‐19 symptoms severity on athletes.

## 5 CONCLUSION

In the present investigation, a group of professional football players reported mild COVID‐19 symptomatology with no need for hospitalization. “General fatigue” and “muscle fatigue” symptoms were the most common long‐term COVID‐19 symptoms reported, and players’ capability to exercise at high intensities was compromised following COVID‐19. These findings collectively suggest that long‐term “general fatigue” and “muscle fatigue” symptoms may play a relevant role in athletes’ physical performance following COVID‐19 and should carefully be considered for a safer and effective RTS.

The spirometry test results indicate that COVID‐19 seems not to alter the pulmonary function of football players with mild symptomatology. However, some substantial reductions found at the individual level demands further investigations.

## AUTHOR CONTRIBUTIONS

Conception or design of the work: Chiara Gattoni, Carlo Capelli and Michele Girardi. Acquisition, analysis or interpretation of data for the work: Chiara Gattoni, Emanuele Conti, Andrea Casolo, Stefano Nuccio, Carmine Baglieri, Carlo Capelli and Michele Girardi. Chiara Gattoni and Michele Girardi wrote the first draft of the manuscript. All the Authors revisited the work critically for important intellectual content. All authors approved the final version of the manuscript and agreed to be accountable for all aspects of the work in ensuring that questions related to the accuracy or integrity of any part of the work are appropriately investigated and resolved. All persons designated as authors qualify for authorship, and all those who qualify for authorship are listed.

## CONFLICT OF INTEREST

The authors declare that they have no conflict of interest.

## ETHICS STATEMENT

This study was reviewed and approved by the Ethics Committee from the University of Essex (ETH2122‐0111), and all the procedures were conducted in conformity with the Declaration of Helsinki. All players signed a written informed consent and agreed to share their data for research purposes. All the tests and procedures used were in accordance with the COVID‐19 RTS guidelines established by the Medical Commission of the Italian Football Federation.
